# A novel polyadenylation signal variant NM_000517.6 (*HBA2*): c.*92_*97delinsTA causing α-thalassemia in two Chinese families

**DOI:** 10.3389/fmed.2026.1766377

**Published:** 2026-03-31

**Authors:** Weirong Huang, Xiaohong Lai, Ningke Zhang, Yajun Su, Yuqing Yang, Hua Wei, Shurong Hong

**Affiliations:** Department of Molecular Genetic Center, Zhangzhou Municipal Hospital Affiliated to Fujian Medical University, Zhangzhou, China

**Keywords:** DNA sequencing, *HBA2*, novel mutation, poly(A) site, α-thalassemia

## Abstract

**Introduction:**

Thalassemia, the most prevalent recessive genetic disorder in China, predominantly affects the southern coastal regions. The mutational spectrum of α-thalassemia (α-thal) continues to expand with advances in detection technologies.

**Methods:**

Fetal DNA was extracted from amniotic fluid samples of the pregnant woman in Family 1 via amniocentesis, and next-generation sequencing (NGS) was used to detect the genetic variant in the participants.

**Results:**

We report a novel *HBA2* mutation [NM_000517.6 (*HBA2*): c.*92_*97delinsTA] in the polyadenylation [poly(A)] site identified in two unrelated Chinese families. The proband’s father and paternal grandmother in Family 1 and a pregnant woman in Family 2 exhibited hematological phenotypes. NGS confirmed that all four individuals were heterozygous carriers of NM_000517.6(HBA2):c.*92_*97delinsTA.

**Discussion:**

This novel variant expands the genetic spectrum of α-thalassemia.

## Introduction

Thalassemia is one of the most common hereditary disorders worldwide. As evidenced by comprehensive epidemiological investigations, thalassemia carrier frequency displays substantial geographic variation across global regions. On a worldwide scale, roughly 5% carry α-thalassemia variants that result in either asymptomatic status or mild clinical phenotypes (1). The prevalence of thalassemia follows the predicted pattern of being higher in the Middle East, Asia, and Mediterranean than in Europe or North America (2–4). Normal individuals have four functional α-globin genes. Each copy of chromosome 16 (16p13.3) contains one α2-globin gene (*HBA2*) and one α1-globin gene (*HBA1*). In adults, the predominant hemoglobin is Hb A (α_2_β_2_), composed of two α- and two β-globin chains (5). The clinical manifestations of α-thalassemia range from no symptoms to severe transfusion-dependent anemia. Deletions of one or two α-globin genes typically result in the α-thalassemia trait, which is usually mild or asymptomatic. In contrast, Hb H disease (6), caused by the inactivation of three α-globin genes, presents with an intermediate severity. This poly(A) site mutation within the 3′-UTR of *HBA2* does not alter the amino acid sequence of α-globin, but downregulates its protein expression by interfering with mRNA processing and maturation. Therefore, such non-deletional mutations targeting the 3′-UTR poly(A) site destabilize mRNA and impair hemoglobin synthesis (5). This study describes two families with a novel heterozygous nucleotide deletion in the poly(A) site of an α-globin gene, expanding the genetic diversity of α-thalassemia and highlighting the importance of non-deletional mutations in disease severity.

## Materials and methods

### Routine blood test and hemoglobin electrophoresis screening

Peripheral venous blood samples were collected in EDTA anticoagulant tubes during routine clinical visits between November 2023 and October 2024 at Zhangzhou Municipal Hospital Affiliated to Fujian Medical University. Complete blood counts were performed using an automated hematology analyzer (BC7500 Automated Hematology Analyzer, Mindray, Shenzhen, China) to obtain red blood cell indices, including hemoglobin concentration, mean corpuscular volume (MCV), and mean corpuscular hemoglobin (MCH), for preliminary anemia screening.

Hemoglobin fraction analysis was conducted using capillary electrophoresis (fully automated capillary electrophoresis system and its associated reagents, Sebia, Lisses, France). This method was applied as part of the routine diagnostic workflow of the clinical laboratory to separate, identify, and quantify hemoglobin variants and fractions in peripheral blood, enabling initial screening for suspected hemoglobinopathies.

### Detection of common thalassemia genes

Genomic DNA was extracted from peripheral blood leukocytes using standard laboratory procedures. Common α- and β-thalassemia mutations were detected by polymerase chain reaction–reverse dot hybridization (PCR-RDB). This assay targeted 23 common thalassemia genotypes, including three α-thalassemia deletions (–SEA, −α^3.7^, and −α^4.2^), three non-deletional α-thalassemia variants (αCSα, αQSα, αWSα), and 17 β-thalassemia point mutations. The PCR-RDB assay was selected based on its established clinical utility in routine thalassemia carrier screening within our laboratory.

### Genetic sequencing

For cases with inconclusive results or discordance between hematological findings and routine genetic testing, next-generation sequencing (NGS) was performed for comprehensive mutation analysis. Library preparation, sequencing, and bioinformatic analysis were conducted as previously described. The targeted regions included the α- and β-globin gene clusters (*HBA1*, NG_000006.1; *HBA2*, NG_000006.1; *HBB*, NG_000007.3), and the detection panel covered all known pathogenic point mutations and common copy number variations.

Putative pathogenic variants identified by NGS were further validated by Sanger sequencing in probands and available family members following established protocols (5). All laboratory analyses were conducted at the medical test center of BGI Genomics.

### Ethics statement

This study was conducted in accordance with the Declaration of Helsinki and was approved by the Ethics Committee of Zhangzhou Municipal Hospital Affiliated to Fujian Medical University (approval number: 202407191701000277133). Written informed consent was obtained from all participants or their legal guardians prior to sample collection and genetic analysis.

## Case report and results

**FIGURE 1:**
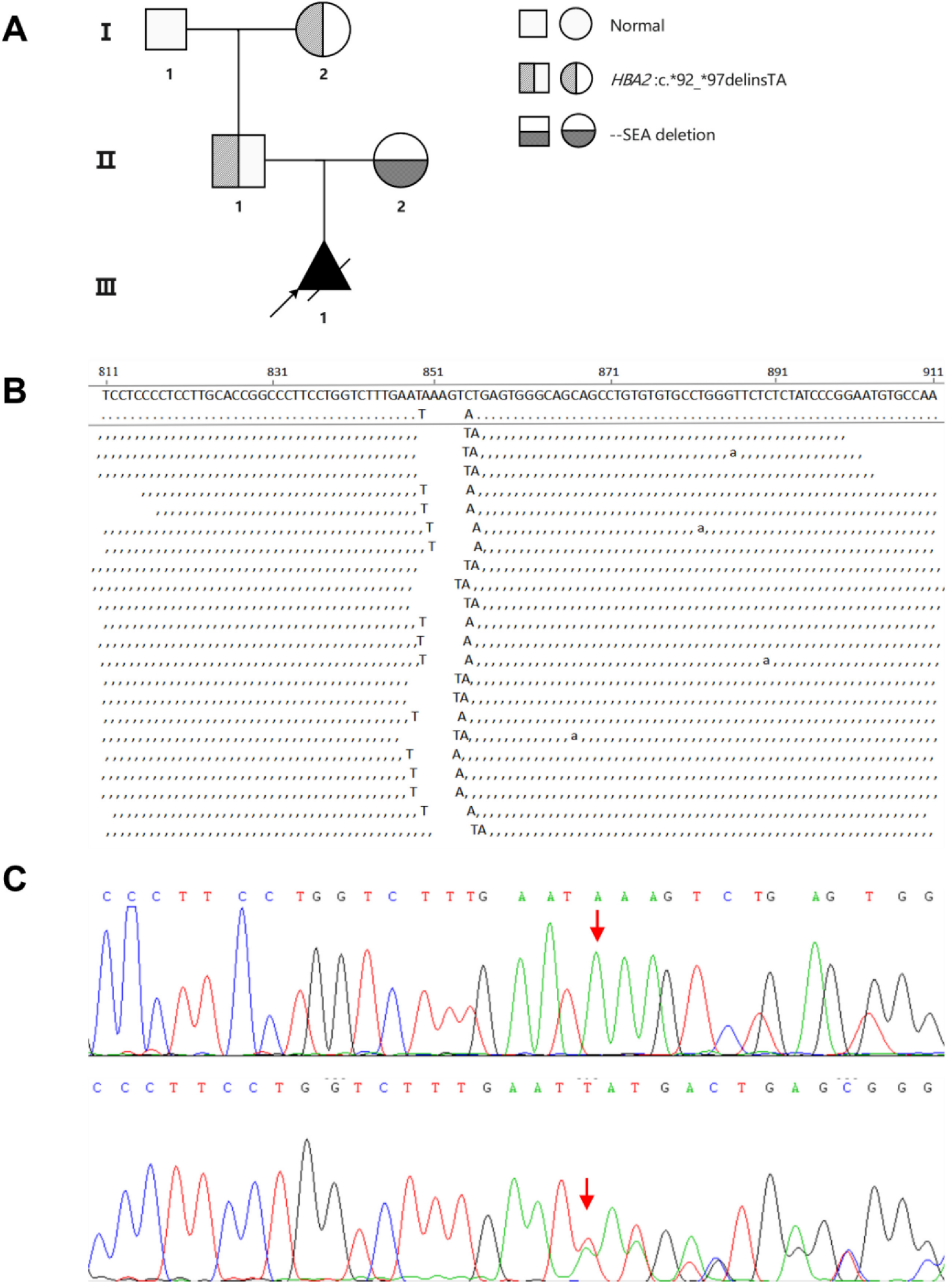
**(A)** Pedigree chart of the case study. **(B)** Next-generation high-throughput sequencing variant map. **(C)** Sanger sequencing was used to validate the chromatogram of the proband.

A 26-year-old pregnant woman at 25 weeks of gestation was found by ultrasound to have a fetus with pericardial effusion, mild tricuspid regurgitation, and elevated middle cerebral artery peak systolic velocity (MCAPSV1.52–1.62 MoM), suggesting moderate to severe fetal anemia. The pregnant woman exhibited microcytic hypochromic anemia. The pregnant woman was identified as a carrier of the Southeast Asian α-thalassemia deletion (αα/–*^SEA^*), while the proband was found to harbor a novel, unreported mutation at the poly(A) site of *HBA2*, which is absent from ClinVar, HbVar, and ITHANET databases. According to the HGVS nomenclature, this mutation was designated as NM_000517.6 (*HBA2*): c.*92_*97delinsTA. To evaluate the pathogenicity of this variant, hematological testing, hemoglobin analysis, and genetic testing were performed on the proband’s parents. The results showed that the father carried the same *HBA2* mutation and had microcytic hypochromia, supporting its pathogenicity (Table 1 and Figure 1).

**TABLE 1:** Hematological and genetic test results for Family 1.

Parameters	Proband (fetus)	Grandfather I-1	Grandmother I-2	Father II-1	Mother II-2
Hb (g/dL)	70	182	131	145	123
RBC (10^12^/L)	3.21	5.81	5.56	5.96	5.39
MCV (fL)	91.6	92.7	70.9	74.3	73.7
MCH (pg)	21.9	31.4	23.6	24.3	22.9
MCHC (g/dl)	244	339	332	327	314
RDW (%)	23.5	12.7	14.5	13.5	16.6
Hb A (%)	13.4	97.5	97.5	97.7	97.5
Hb A_2_ (%)	0	2.5	2.5	2.3	2.5
Hb F (%)	23.1	0	0	0	0
Hb C (%)	0	0	0	0	0
Variant Hb (%)	62.2 (Hb Bart’s)	0	0	0	0
Genotype	α^poly(A) (AAAGTC>TA)^α/- -SEA	αα/αα	α^poly(A) (AAAGTC>TA)^α/αα	α^poly(A) (AAAGTC>TA)^α/αα	αα/- -SEA

**FIGURE 2:**
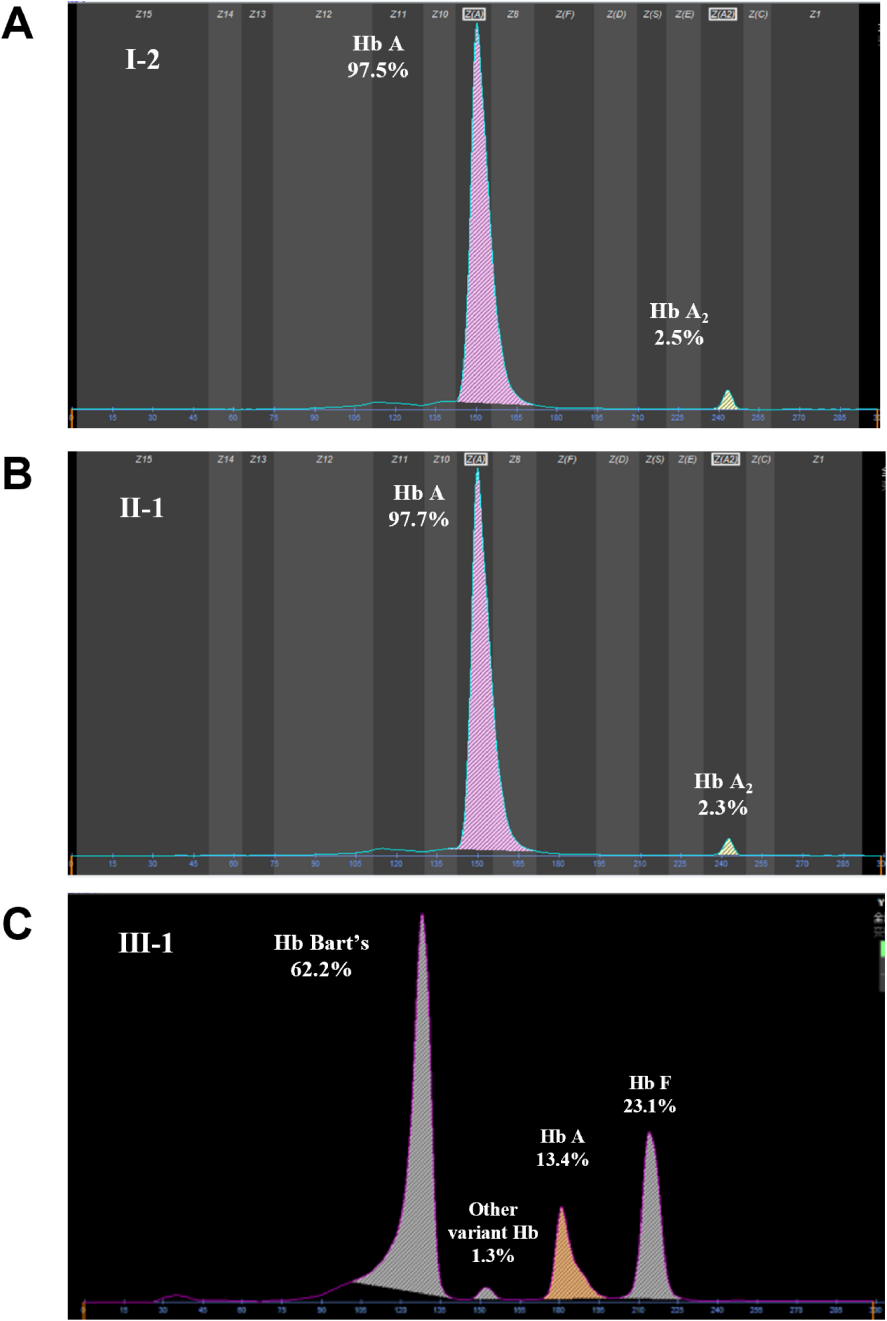
Electrophoretic diagram of **(A)** grandmother (I-2), **(B)** father (II-1) and **(C)** proband (III-1).

At 26 weeks of gestation, ultrasound examination demonstrated rapid deterioration of fetal hydrops, indicative of moderate-to-severe fetal anemia. After counseling, the couple selected pregnancy termination. Cord blood sample collected prior to induction of labor showed 62.2% Hb Bart’s, 23.1% HbF, 13.4% HbA and 1.3% a fast-moving variant, confirming the diagnosis of severe α-thalassemia (Hb Bart’s hydrops fetalis). Molecular analysis demonstrated that the fetus had inherited the maternal deletion and the paternal novel poly(A) mutation [NM_000517.6 (*HBA2*): c.*92_*97delinsTA], establishing biallelic α-globin gene defects as the cause of the phenotype (Figures 2A–C).

**FIGURE 3:**
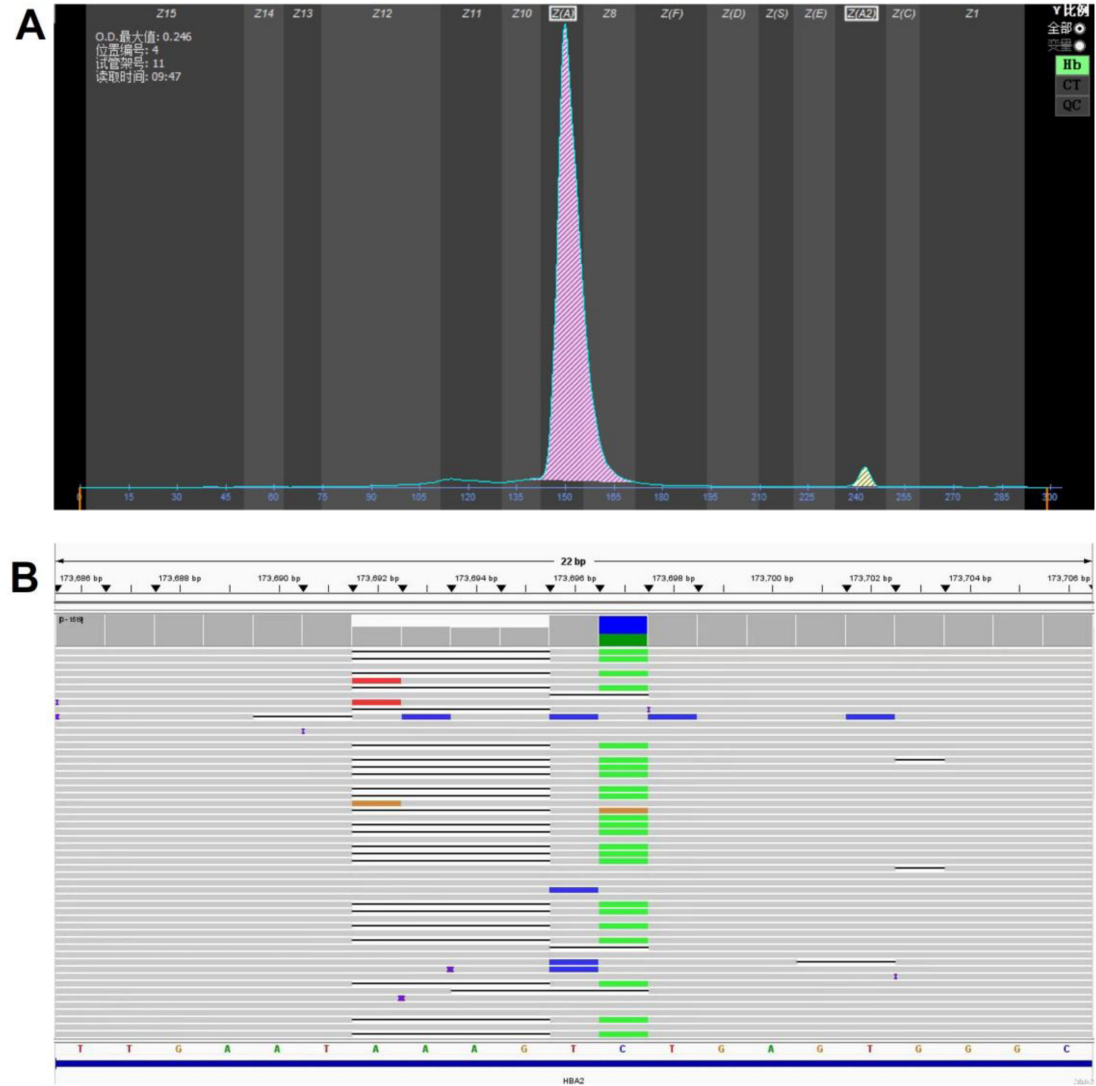
**(A)** Electrophoretic diagram of the proband from Family 2. **(B)** Next-generation high-throughput sequencing variant map.

In the second family, a 24-year-old pregnant woman presented at 30 weeks of gestation with microcytic hypochromic anemia (MCV: 78.1 fL, MCH: 24.5 pg). Hemoglobin electrophoresis showed 2.3% *HBA*_2_ without other variants (Figure 3A), which prompted genetic testing for thalassemia. The NGS testing result was NM_000517.6 (*HBA2*): c.*92_*97delinsTA, which was identical to the pathogenic variant identified in the first case (Figure 3B).

According to the American College of Medical Genetics and Genomics/Association for Molecular Pathology (ACMG/AMP) guidelines for variant pathogenicity classification, this novel *HBA2* variant was classified as a variant of uncertain significance (VUS).

Based on the consistent hematological phenotypes observed in the two Chinese families, we infer that this variant is associated with α^+^-thalassemia.

## Discussion

This study reports a novel *HBA2* poly(A) mutation [NM_000517.6 (*HBA2*): c.*92_*97delinsTA], identified by NGS in two families with thalassemia traits. In Family 1, this variant co-occurred with a maternal –SEA deletion, resulting in severe fetal anemia and hydrops fetalis that required pregnancy termination; in the second family, the identical mutation was detected in a proband with microcytic hypochromic anemia undetectable by conventional thalassemia testing (7). Based on the ACMG/AMP guidelines for variant pathogenicity interpretation and the currently available evidence, this novel *HBA2* variant was categorized as a variant of uncertain significance (VUS). The variant was absent from public population genetic databases and detected in trans with a known pathogenic allele of the *HBA2* gene. Furthermore, it exhibited complete genotype-phenotype co-segregation with mild α^+^-thalassemia phenotypes in all affected individuals. Although these observations collectively support a potential causal association between the variant and the observed hematological phenotypes, the current evidence remains insufficient to classify the variant as likely pathogenic—particularly given its localization within the non-coding region, where the potential impact on gene expression and protein function cannot be fully elucidated without additional RNA-level validation.

The NM_000517.6 (*HBA2*): c.*92_*97delinsTA mutation localizes to the core AAUAAA poly(A) signal region in the 3′ untranslated region (3′-UTR), a highly conserved regulatory element governing eukaryotic mRNA maturation. Sequence variants disrupting this canonical motif are well-documented to impair α-globin gene mRNA maturation, and known pathogenic *HBA2* 3′-UTR poly(A) site variants—such as c.*91_*92delTA—have been validated to abrogate canonical mRNA 3′ polyadenylation, causing impaired nuclear export and reduced mRNA stability (5, 8). In the present study, the co-occurrence of this variant with microcytic hypochromic anemia in affected patients strongly supports the proposition that this molecular process contributes functionally to the observed clinical phenotype. Collectively, these findings indicate that the NM_000517.6 (*HBA2*): c.*92_*97delinsTA mutation disrupts the canonical poly(A) signal, thereby compromising mRNA post-transcriptional processing and reducing α-globin synthesis, which potentially drives mild α^+^-thalassemia in heterozygous carriers. Furthermore, when compounded with a large α-globin deletion (–SEA), this variant may exacerbate α-globin chain imbalance, ultimately leading to the severe fetal thalassemia observed in the first family.

This variant’s functional effects align with well-characterized pathogenic *HBA2* poly(A) site mutations, further supporting that 3′-UTR poly(A) signal disruption contributes to α^+^-thalassemia pathogenesis. Its identification also highlights NGS’s critical advantage over traditional testing, which lacks sensitivity for rare or novel non-coding region variants (9). Clinically, the severe fetal phenotype in the first family underscores the potential reproductive risk of this variant when trans-combined with common α-thalassemia deletions. For couples with abnormal hematological indices in thalassemia-prevalent populations, comprehensive NGS-based genetic testing and pre-pregnancy counseling are essential to assess potential reproductive risks and prevent adverse perinatal outcomes.

This study expands the spectrum of *HBA2* variants associated with α-thalassemia and provides functional insights into how poly(A) signal region variants may disrupt α-globin gene expression, while reinforcing NGS’s value for detecting rare/novel non-coding variants missed by conventional methods (10). In accordance with ACMG/AMP guidelines, this variant is classified as a variant of uncertain significance (VUS). Although the clinical, genetic, and contextual findings are consistent with a potential pathogenic role, no definitive functional evidence is available in the present study to confirm pathogenicity. Further investigations in larger cohorts and additional functional studies are needed to clarify the clinical relevance and definitive pathogenicity of this variant.

Limitations of the present study include the lack of *in vitro* functional assays to further verify the regulatory effect of this mutation on polyadenylation efficiency, and the small sample size of the studied families. Future studies with larger cohorts and more comprehensive functional experiments are needed to validate the pathogenicity and clinical relevance of this variant.

## Data Availability

The data that support the findings of this study are available from the corresponding author upon reasonable request.

## References

[1] Kattamis A Kwiatkowski J Taher A Musallam K Cappellini M Porter J et al. Epidemiology of clinically significant forms of alpha- and beta-thalassemia: a global map of evidence and gaps. *Blood Rev.* (2024) 56:100998. 10.1002/ajh.27006 37357829

[2] GBD 2021 Hemoglobinopathies and Hemolytic Anemias Collaborators, Ojo TT Amegbor PM Islam F Gyamfi J Mai A et al. Burden of hemoglobinopathies and hemolytic anemias in the World Health Organization African region, 2000-2021: findings from the Global Burden of Disease 2021 study. *PLoS Glob Public Health.* (2025) 5:e0005197. 10.1371/journal.pgph.0005197 40982566 PMC12453249

[3] Modell B Darlison M. Global epidemiology of haemoglobin disorders and derived service indicators. *Bull World Health Organ.* (2008) 86:480–7. 10.2471/blt.06.036673 18568278 PMC2647473

[4] Musallam K Lombard L Kistler K Arregui M Gilroy K Chamberlain C et al. Epidemiology of clinically significant forms of alpha- and beta-thalassemia: a global map of evidence and gaps. *Am J Hematol.* (2023) 98:1436–51. 10.1002/ajh.27006 37357829

[5] Forouzesh Pour F Karimi K Ghaderi Z Tavakoli Koudehi A Najmabadi H. Heterozygosity for the Novel *HBA2*: c.*91_*92delTA polyadenylation site variant on the α2-globin gene expanding the genetic spectrum of α-thalassemia in Iran. *Hemoglobin.* (2020) 44:423–6. 10.1080/03630269.2020.1831529 33054440

[6] Lal A Vichinsky E. The clinical phenotypes of alpha thalassemia. *Hematol Oncol Clin North Am.* (2023) 37:327–39. 10.1016/j.hoc.2022.12.004 36907606

[7] Shang X Peng Z Ye Y Asan, Zhang X Chen Y et al. Rapid targeted next-generation sequencing platform for molecular screening and clinical genotyping in subjects with hemoglobinopathies. *EBioMedicine.* (2017) 23:150–9. 10.1016/j.ebiom.2017.08.015 28865746 PMC5605365

[8] Chen M Ye Y Liao L Deng X Qiu Y Lin F. Hereditary spherocytosis overlooked for 7 years in a pediatric patient with β-thalassemia trait and novel compound heterozygous mutations of SPTA1 gene. *Hematology.* (2020) 25:438–45. 10.1080/16078454.2020.1846874 33210974

[9] Zhang Q Fan X Xu M Zhang Y Xu H Wen X et al. Hb H disease caused by multiple mutations in the polyadenylation signal site and - -(SEA)/αα. *Hemoglobin.* (2017) 41:189–92. 10.1080/03630269.2017.1366917 28950779

[10] Pan L Tian P Chen S Zhang R. Novel promoter mutation (HBB:C.-139_-138del) associated with β-thalassemia trait detected by next-generation sequencing in Southern China. *Hemoglobin.* (2023) 47:21–4. 10.1080/03630269.2023.2182215 36866928

